# Supplementary material for: Enteric Pathogens Detected in Children under Five Years Old Admitted with Diarrhea in Moshi, Kilimanjaro, Tanzania
Source: Pathogens. 2023 Apr 19;12(4):618. doi: 10.3390/pathogens12040618 (PMC10143310; doi:10.3390/pathogens12040618)
Supplement: Supplementary file 1 [file pathogens-12-00618-s001.zip › pathogens-2248824-supplementary.pdf]

**Supplementary materials**

**Table S1. TAC pathogen targets, primer-probe sequences and CT cut-offs for diarrhea associated pathogens**

| **Pathogen** | **Target** | **Primers & Probe sequences**<br>**(Forward primer-F, Reverse primer-R, Probe-P)** | **CT for diarrhea associated*** | **Ref** |
|---|---|---|---|---|
| Adenovirus 40/41 | Fiber gene | F: AACTTTCTCTCTTAATAGACGCC<br>R: AGGGGGCTAGAAAACAAAA<br>P: CTGACACGGGCACTCT | 35.0 | [1] |
| Astrovirus | Capsid | F: CAGTTGCTTGCTGCGTTCA<br>R: CTTGCTAGCCATCACACTTCT<br>P: CACAGAAGAGCAACTCCATCGC | 25.5 | [1] |
| Norovirus GI | ORF1-2 | F: CGYTGGATGCGNTTYCATGA<br>R:CTTAGACGCCATCATCATTYAC<br>P:TGGACAGGAGATCGC | | [1] |
| Norovirus GII | ORF1-2 | F: AGGATCCATTGCAAGAGGG<br>R: CTACATCAAGCGTGGATGGC<br>P: TGGTCAGTTGGTACCGGAG | 27.6 | [1] |
| Rotavirus | *NSP3* | F:ACCATCTWCACRTRACCCTCTATGAG<br>R: GGTCACATAACGCCCCTATAGC<br>P:AGTTAAAAGCTAACACTGTCAAA | 35.0 | [1] |
| Sapovirus | *RdRp* | F: GAYCASGCTCTCGCYACCTAC<br>F: TTGGCCCTCGCCACCTAC<br>R: CCCTCCATYTCAAACACTA<br>P: CCRCCTATRAACCA | 31.6 | [1] |
| EAEC | *aaiC* | F: ATTGTCCTCAGGCATTTCAC<br>R:ACGACACCCCTGATAAACAA<br>P: TAGTGCATACTCATCATTTAAG | | [1] |
| EAEC | *aatA* | F: CTGGCGAAAGACTGTATCAT<br>R: TTTTGCTTCATAAGCCGATAGA<br>P:TGGTTCTCATCTATTACAGACAGC | | [1] |
| EPEC | *eae* | F: CATTGATCAGGATTTTTCTGGTGATA<br>R: CTCATGCGGAAATAGCCGTTA<br>P: ATACTGGCGAGACTATTTCAA | | [1] |
| EPEC | *bfpA* | F: TGGTGCTTGCGCTTGCT<br>R: CGTTGCGCTCATTACTTCTG<br>P: CAGTCTGCGTCTGATTCCAA | 19.5 | [1] |
| ETEC | *LT* | F: TTCCCACCGGATCACCAA<br>R: CAACCTTGTGGTGCATGATGA<br>P: CTTGGAGAGAAGAACCCT | | [1] |
| ETEC | *STh*<br><br>*STp* | F: GCTAAACCAGYAGRGTCTTCAAAA<br>F: CCCGGTACARGCAGGATTACAACA<br>R: TGGTCCTGAAAGCATGAA | 26.2 | [1] |

| | | | | |
|---|---|---|---|---|
| | | R: TGAATCACTTGACTCTTCAAAA<br>P: GGCAGGATTACAACAAAGTT<br>P: TGAACAACACATTTTACTGCT | | |
| STEC | *stx1* | F: ACTTCTCGACTGCAAAGACGTATG<br>R: ACAAATTATCCCCTGWGCCACTATC<br>P: CTCTGCAATAGGTACTCCA | | [1] |
| STEC | *Stx2* | F: CCACATCGGTGTCTGTTATTAACC<br>R: GGTCAAAACGCGCCTGATAG<br>P: TTGCTGTGGATATACGAGG | | [1] |
| Aeromonas | *Aerolysin* | F: TYCGYTACCAGTGGGACAAG<br>R: CCRGCAAACTGGCTCTCG<br>P: CAGTTCCAGTCCCACCACTT | | [1] |
| *Campylobacter* | *cpn60* | F: AAAGTIGGMAAAGATGGTGTTAT<br>F: AAAGTIGGWAAAGACGGYGTTAT<br>R: TCAAATTGCATACCYTCAAC<br>P: TTTGCCTCTTCMACAGT<br>P: TTTGCTTCTTCWACAGT | | [1] |
| *Campylobacter jejuni* | *hipO* | F: CTTGCGGTCATGATGGACATAC<br>R: AGCACCACCCAAACCCTCTTCA<br>P: TGCTTGCTGCAAAGTATT | 19.7 | [1] |
| *Campylobacter coli* | *GlyA* | F: AAACCAAAGCTTATCGTGTGC<br>R: AGTGCAGCAATGTGTGCAAT<br>P: TAAGCTCCAACTTCATCCG | | [1] |
| *Clostridium difficile* | *tcdA* | F: TTCAAGCAGAAATAGAGCACTC<br>R: TATCAGCCCATTGTTTTATGTATTC<br>P: CACTGACTTCTCCACCTATCCA | | [1] |
| | *tcdB* | F: GGTATTACCTAATGCTCCAAATAG<br>R: TTTGTGCCATCATTTTCTAAGC<br>P: CCTGGTGTCCATCCTGTTTC | | [1] |
| *Cryptosporidium* | 18S rRNA | F: GGGTTGTATTTATTAGATAAAGAACCA<br>R: AGGCCAATACCCTACCGTCT<br>P: TGACATATCATTCAAGTTTCTGAC | 29.1 | [1] |
| *Cyclospora cayetanensis* | 18S rRNA | F: AAAAGCTCGTAGTTGGATTTCTG<br>R: AACACCAACGCACGCAGC<br>P: AAGGCCGGATGACCACGA | | [1] |
| *Helicobacter pylori* | *ureC* | F: GACACCAGAAAAAGCGGCTA<br>R: AGCGCATGTCTTCGGTTAAA<br>P: TCACTAAAGCGTTTTCTACC | 30.8 | [1] |
| *Cystoisospora belli* | 18S rRNA | F: ATATTCCCTGCAGCATGTCTGTTT<br>R: CCACACGCGTATTCCAGAGA<br>P: CAAGTTCTGCTCACGCGCTTCTGG | | [1] |
| *Mycobacterium tuberculosis* | IS6110 | F: GGGTAGCAGACCTCACCTATG<br>R: AGCGTAGGCGTCGGTGA<br>P: TCGCCTACGTGGCCTTT | | [1] |
| *Salmonella enterica* | *ttr* | F: CTCACCAGGAGATTACAACATGG<br>R: AGCTCAGACCAAAAGTGACCATC<br>P: CACCGACGGCGAGACCGACTTT | 32.4 | [1] |

| | | | | |
|---|---|---|---|---|
| *Shigella*/enteroinvasive *E. coli* (EIEC) | *ipaH* | F: CCTTTTCCGCGTTCCTTGA<br>R: CGGAATCCGGAGGTATTGC<br>P: CGCCTTTCCGATACCGTCTCTGCA | 33.1 | [1] |
| *Shigella flexneri* | *Putative periplasmic protein* | F: TGGGTGCATCCTGACCTGT<br>R: GACAAACAATAACGAGCTACCGAT<br>P: ACCACGGAATAATCCCGCAG | | [2] |
| | *O-antigen#* | F: CTCCTATCCGTGATTATAGTGCA<br>R: GCACACACAACTCACTGTATTT<br>P: TCCTTCTCACGATTAAAATC | | [2] |
| | *Type 3 restriction enzyme#* | F: CTTTCAACGCACGAATATCAAC<br>R: GAACCTGATCCAGACGGAGA<br>P: TTCTTCAGAACCGGGTTTTG | | [2] |
| *Shigella sonnei* | *Putative methylase* | F: TGCCGCTAAAATCCTTCTGT R:<br>GCGTACGACGAAAGGAAAAA<br>P: GAAGTTATTGATTCCGCCC | | [2] |
| *Plesiomonas shigelloides* | *gyrB* | F: CCGCCGTGAAGGCAAAG<br>R: GCTACCGGCTCACCCAGAT<br>P: CACACCCAAGAATAC | | [1] |
| *Vibrio cholerae* | *hlyA* | F: ATCGTCAGTTTGGAGCCAGT<br>R: TCGATGCGTTAAACACGAAG<br>P: ACCGATGCGATTGCCCAA | 34.9 | [1] |
| *Enterocytozoon bieneusi* | *ITS* | F: CACCAGGTTGATTCTGCCTGAC<br>R: CTAGTTAGGCCATTACCCTAACTACCA<br>P: CTATCACTGAGCCGTCC | | [1] |
| *Encephalitozoon intestinalis* | SSU rRNA | F: TGTGTAGGCGTGAGAGTGTATCTG<br>R: CATCCAACCATCACGTACCAATC<br>P: CACTGCACCCACATCCCTCACCCTT | | [1] |
| *Entamoeba histolytica* | 18S rRNA | F: ATTGTCGTGGCATCCTAACTCA<br>R: GCGGACGGCTCATTATAACA<br>P: TCATTGAATGAATTGGCCATTT | | [1] |
| *Giardia lamblia* | 18SrRNA | F: GACGGCTCAGGACAACGGTT<br>R: TTGCCAGCGGTGTCCG<br>P: CCCGCGGCGGTCCCTGCTAG | | [1] |
| *Ancylostoma duodenale* | *ITS2* | F: GAATGACAGCAAACTCGTTGTTG<br>R: ATACTAGCCACTGCCGAAACGT<br>P: ATCGTTTACCGACTTTAG | | [1] |
| *Ascaris lumbricoides* | *ITS1* | F: GCCACATAGTAAATTGCACACAAAT<br>R: GCCTTTCTAACAAGCCCAACAT<br>P:TTGGCGGACAATTGCATGCGAT | | [1] |
| *Necator americanus* | *ITS2* | F: CTGTTTGTCGAACGGTACTTGC<br>R: ATAACAGCGTGCACATGTTGC<br>P: CTGTACTACGCATTGTATAC | | [1] |
| *Strongyloides stercoralis* | Dispersed repetitive sequence | F: TCCAGAAAAGTCTTCACTCTCCAG<br>R:TGCGTTAGAATTTAGATATTATTGTTGCT<br>P: TCAGCTCCAGTTGAACAACAGCCTCCAA | | [1] |
| *Trichuris trichiura* | 18S rRNA | F: TTGAAACGACTTGCTCATCAACTT<br>R: CTGATTCTCCGTTAACCGTTGTC | | [1] |

| | | | | |
|---|---|---|---|---|
| | | P: CGATGGTACGCTACGTGCTTACCATGG | | |
| MS2 | *MS2gl* | F: TGGCACTACCCCTCTCCGTATTCAC<br>R: GTACGGGCGACCCCACGATGAC<br>P: CACATCGATAGATCAAGGTGCCTACAAGC | | [1] |
| PhHV | *gB* | F: GGGCGAATCACAGATTGAATC<br>R: GCGGTTCCAAACGTACCAA<br>P: TATGTGTCCGCCACCATCT | | [1] |

*This assay detects most *S. flexneri* serotypes except for serotype 6.

#The combination of these two assays identifies *S. flexneri* serotype 6 when both are positive (Cq≤35).

**References**


1. Liu J, Gratz J, Amour C, Nshama R, Walongo T, Maro A, et al. Optimization of quantitative PCR methods for enteropathogen detection. PLoS One. 2016;11: 1–11. doi:10.1371/journal.pone.0158199

2. Liu J, Platts-Mills JA, Juma J, Kabir F, Nkeze J, Okoi C, et al. Use of quantitative molecular diagnostic methods to identify causes of diarrhoea in children: a reanalysis of the GEMS case-control study. Lancet. 2016;388: 1291–1301. doi:10.1016/S0140-6736(16)31529-X


*Effect of co-infection on the clinical presentation of under five years children admitted with diarrhea*

The univariate analysis comparing the effect of co-infection on the clinical presentation of the ill children at admission shows that those with co-infection were two times more likely to present with flatulence compared to those with no or mono-infection IRR; 95%CI = 2.42 (1.11-5.42). After adjusting for other clinical symptoms, co-infection was significantly associated with flatulence and loss of appetite as compared to those with no or mono-infection IRR; 95%CI = 2.24 (1.09-4.58) and 1.81 (1.06 – 3.08) respectively. Further, those with running nose were less likely to have co-infection IRR; 95%CI = 0.50 (0.28-0.89). No statistical significance was observed in other clinical presentations. More details are found in the table below

Table S2. Univariate and multivariable analysis of effect of co-infection by diarrhea associated pathogens on the clinical presentation of hospitalized children (N=146).

| Clinical Symptoms | Co-infection | | Crude IRR | Adjusted IRR (95% CI) | P-value |
|---|---|---|---|---|---|
| | No (N=108) | Yes (N=38) | | | |
| **No: -_stools in 24hr** | | | | | |
| 3-5 | 62 (75.61) | 20 (24.39) | Ref | | |
| ≥6 | 46 (71.88) | 18 (28.13) | 1.15 (0.67-1.99) | - | |
| Nausea | | | | | |
| No | 104 (74.29) | 36 (25.71) | Ref | - | |
| Yes | 4 (66.67) | 2 (33.33) | 1.29 (0.40-4.18) | - | |
| Vomiting | | | | | |
| No | 13 (76.47) | 4 (23.53) | Ref | | |
| Yes | 95 (73.64) | 34 (26.36) | 1.12 (0.45 – 2.77) | - | |

| | | | | | |
|---|---|---|---|---|---|
| Abdominal Pain | | | | | |
| No | 103 (73.57) | 37 (26.43) | Ref | | |
| Yes | 5 (83.33) | 1 (16.67) | 0.63 (0.10– 3.88) | - | |
| Fever | | | | | |
| No | 43 (74.14) | 15 (25.86) | Ref | | |
| Yes | 65 (73.86) | 23 (26.14) | 1.00 (0.74 – 1.36) | - | |
| Fatigue | | | | | |
| No | 101 (75.37) | 33 (24.63) | Ref | | |
| Yes | 7 (58.33) | 5 (41.67) | 1.69 (0.81 – 3.53) | - | |
| Flatulence | | | | | |
| No | 106 (75.18) | 35 (24.82) | | Ref | |
| Yes | 2 (40.00) | 3 (60.00) | 2.42 (1.11 – 5.24) | 2.24 (1.09-4.58) | 0.028 |
| Running nose | | | | | |
| No | 54 (67.50) | 26 (32.50) | Ref | Ref | |
| Yes | 54 (81.82) | 12 (18.18) | 0.56 (0.31- 1.02) | 0.5 (0.28-0.89) | 0.019 |
| Coughing | | | | | |
| No | 56 (70.00) | 24 (30.00) | Ref | | |
| Yes | 52 (78.79) | 14 (21.21) | 0.71 (0.39-1.25) | - | |
| Loss of appetite | | | | | |
| No | 63 (79.75) | 16 (20.25) | Ref | Ref | |
| Yes | 45 (67.16) | 22 (32.84) | 1.62 (0.93-2.83) | 1.81 (1.06-3.08) | 0.030 |

In this study, we found that co-infections were significantly high among children with flatulence and loss of appetite as compared to no or mono-infection, and lower in those with running nose. After adjusting for other clinical features, flatulence, running nose and loss of appetite remained statistically significant associated with confections. However, there were no further evaluations to compare the diarrhea severity among the cases, hence cannot be considered as a criterion for existence of co-infection.
